# Parastomal hernia containing the stomach—a rare complication

**DOI:** 10.1093/jscr/rjad204

**Published:** 2023-06-19

**Authors:** Chris Bodimeade, Rebekka Troller

**Affiliations:** East Surrey Hospital, Redhill, UK; Medway Maritime Hospital, Gillingham, UK

## Abstract

A lady in her 70s presented to hospital with sudden onset nausea and excessive vomiting. She had a constant and worsening abdominal pain that radiated to the back but was focused on her stoma in the left iliac fossa. The patient had bilateral hernias and colostomy following a Hartman’s procedure for perforated diverticulosis in 2018 and had presented twice before in the last 6 months with similar symptoms. CT abdomen pelvis showed a large portion of the stomach in the parastomal hernia leading to a narrowing of the stomach at the hernia neck but no ischaemic changes. She was diagnosed with bowel obstruction and successfully treated with fluid resuscitation, proton pump inhibitors, analgesia, antiemetics and decompression of the stomach using large bore nasogastric tube. A total of 2600 ml fluid was aspirated in 24 h and her stoma restarted normal output. After 10 days she was discharged home.

## INTRODUCTION

Parastomal hernias are relatively common late complications of parastomal hernia; however, it is unusual to have such an established hernia including the stomach. There are only 13 previously documented cases describing a parastomal hernia containing the stomach available in the literature since 1967 [1–13]. This condition is of note to the wider surgical community because of its scarcity and as the patient presented multiple times with the same condition.

## CASE PRESENTATION

A lady in her 70s came to hospital in an ambulance following a 12-h history of abdominal pain, dark vomit and reduced output from her stoma. In 2018, she had a Hartmans operation following perforated diverticular disease leading to a colostomy, her other medical history included diagnoses of high body mass index, right eye blindness, hypertension and osteoporosis. The patient lived independently in her own house, which was noted to be untidy and the patient was unkempt.

Upon arrival in the emergency department, she was in extreme pain, hypoxic and with two large hernias on either side of her abdomen. She was initially treated with IV Co-amoxiclav, IV morphine and antiemetics cyclizine and ondansetron. Admission blood results showed an elevated Lactate of 2.2 but unremarkable inflammatory markers and other biochemistry results (WCC 10.1, CRP 12.5, Plt 425, Crea 50, Egfr > 90, Na 137, K 4.1). She was referred to the surgeons.

On examination by the general surgery on-call team, she was found to have a large body habitus with a soft abdomen, which was tender on the left side around the stoma. There were two prominent hernias. A large parastomal hernia on the left was the source of tenderness, but the stoma was otherwise healthy with reduced content in the bag as reported by the patient. There was also a large soft non-tender hernia on the right side of the abdomen.

Differentials diagnoses considered included incarcerated small bowel hernia, which is a common presentation in parastomal hernias, but this was ultimately disproven by the cross-sectional imaging. A parastomal infection was considered; however, clinically, neither the stoma nor the hernia appeared infected and there was a lack of raised inflammatory markers in the blood tests. Finally, a necrotic stoma could have caused this presentation; however, the stoma appeared pink and healthy throughout admission.

**Figure 1:**
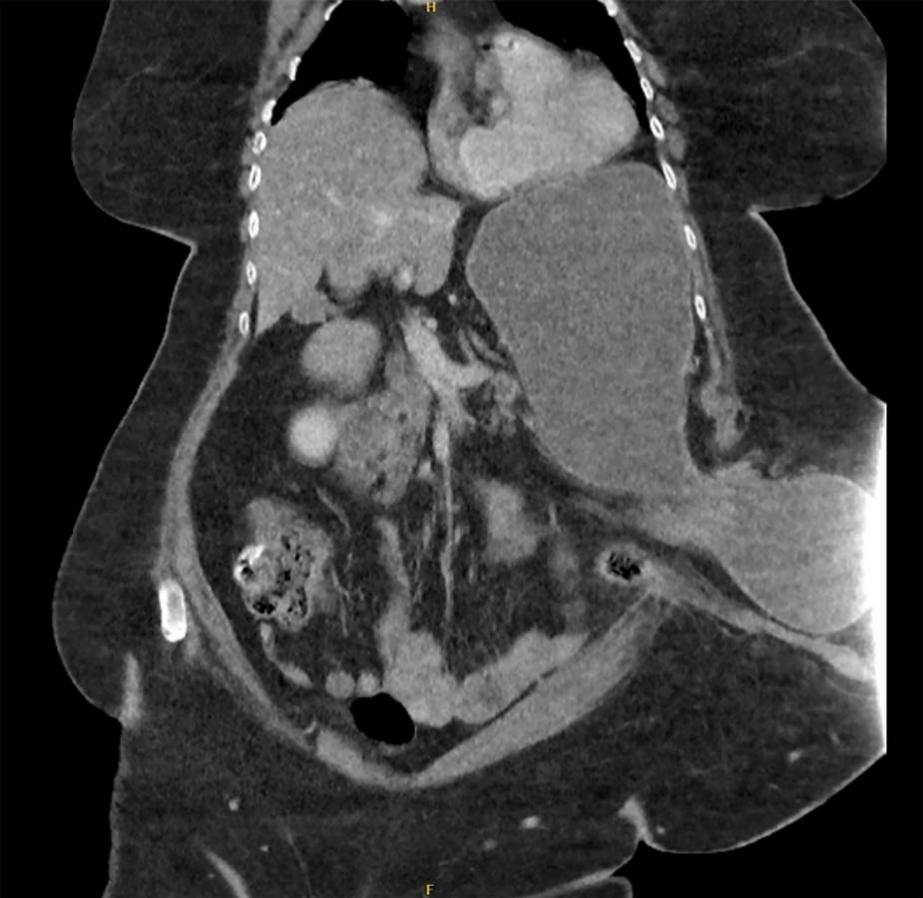
CT abdomen pelvis—coronal view of parastomal hernia containing stomach.

**Figure 2:**
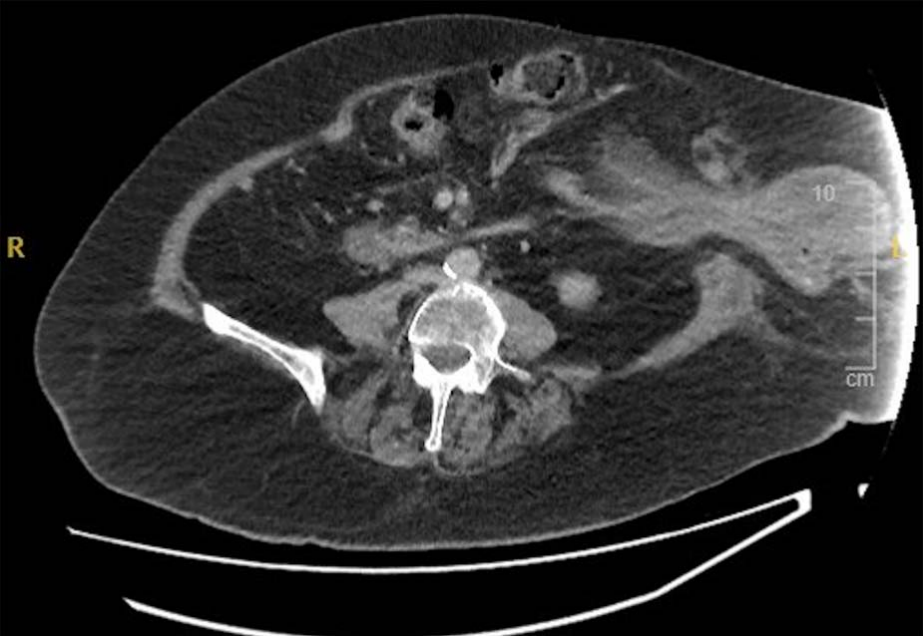
CT abdomen pelvis—axial view of parastomal hernia containing stomach.

The patient underwent a CT abdomen pelvis (Figs 1 and 2), which reported bowel obstruction secondary to a parastomal hernia on the left containing a large portion of the stomach’s body and antrum.

The patient was successfully treated conservatively with fluid resuscitation, antiemetics, analgesia, proton pump inhibitors. Decompression of the herniated stomach was achieved using large bore 14 Gauge nasogastric tube (NGT), 2600 ml of dark stomach content was removed within 24 h. After 4 days the output from the NGT was minimal, it was clamped and then removed. The patients’ stoma output increased and her bloods results normalized. On the 5th day, she was deemed medically fit for discharge. However, she was not able to be discharged until occupational therapy had initiated a package of care and delivered the relevant equipment was developed to her house.

The patient was noted to have had two previous admissions for similar presentations the first 6 months earlier and the second in 10 days prior to the admission described. On both earlier occasions, stomach herniation around the stoma was reported on CT scan and was successfully treated conservatively. On every admission, surgical intervention was considered but deemed to be too high risk because of the patients’ body habitus and ultimately not necessary as stomach decompression was sufficient to reduce the hernia.

## DISCUSSION

Although parastomal hernias are commonly encountered complications after stoma formation, herniation of the stomach around the stoma is rare. There are 12 documented case reports detailing a total of 13 patients with this particular presentation since 1967. The cases overwhelmingly record the condition in women (*n* = 12) and are more commonly treated surgically (*n* = 10). Including our case, three patients were treated conservatively. Although there is a trend in the literature for surgery, it is important to highlight the feasibility of conservative management in some high-risk cases. In the patient described, the options for surgery would have been more likely if we had observed alarming signs of perforation, ischaemia or necrosis in either the stomach or the stoma. However, without those factors, conservative management was preferential given her complex anatomy. Our patient was the only one who has been documented as being successfully treated conservatively on multiple occasions.

## CONCLUSION

- Parastomal hernias are common, in rare instances they can contain the stomach.
- There have been only 13 previously documented cases in the academic literature of parastomal hernias containing the stomach.
- Conservative management of stoma hernias is a viable treatment option in appropriate patients despite a preference for surgery in historical cases.

## EVIDENCE SEARCH

Parastomal hernia involving stomach, Tom Roper, Librarian (5th December, 2022). REDHILL, UK: Surrey and Sussex Library and Knowledge Services.
